# Microbial Ecology in Anaerobic Digestion at Agitated and Non-Agitated Conditions

**DOI:** 10.1371/journal.pone.0109769

**Published:** 2014-10-14

**Authors:** Zhuoli Tian, Léa Cabrol, Gonzalo Ruiz-Filippi, Pratap Pullammanappallil

**Affiliations:** 1 Department of Agricultural and Biological Engineering, University of Florida, Gainesville, Florida, United States of America; 2 Escuela de Ingeniería Bioquímica, Pontificia Universidad Catolica de Valparíaso, General Cruz 34, Valparaíso, Chile; Oak Ridge National Laboratory, United States of America

## Abstract

To investigate the distribution and dynamics of microbial community in anaerobic digestion at agitated and non-agitated condition, 454 pyrosequencing of 16s rRNA was conducted. It revealed the distinct community compositions between the two digesters and their progressive shifting over time. Methanogens and syntrophic bacteria were found much less abundant in the agitated digester, which was mainly attributed to the presence of bacterial genera *Acetanaerobacterium* and *Ruminococcus* with relatively high abundance. The characterization of the microbial community corroborated the digestion performance affected at the agitated condition, where lower methane yield and delayed methane production rate were observed. This was further verified by the accumulation of propionic acid in the agitated digester.

## Introduction

The divergent effect of agitation on anaerobic digestion has been reported by some studies, while most of which investigated the conventional physiochemical properties[1], [2], few evaluated the link between digestion performance and microbial activities, focusing specifically on a small group of bacteria and archaea [3]. However, the ecophysiological role of a great variety of microbes that participate in anaerobic digestion has yet to be fully understood.

Classically, environmental microbial communities are analyzed by construction of 16S rRNA clone libraries and the subsequent sequencing of individual clones. The approach, termed Sanger sequencing, has been applied in Tian et al (2013) to compare the microbial community structures for anaerobic digestion at agitated and non-agitated condition, led to the identification of some major microbial phylotypes in bacteria or archaea domain. Due to the fact that a few numbers of clones can be affordably sequenced, Sanger method has its limitation in revealing the whole complexity of microbial communities and is unlikely to adequately represent the genetic diversity. New development of high-throughput next-generation sequencing technologies (NGS), such as 454 barcoded pyrosequencing, not only eliminates the laborious step of preparing clone libraries, but also makes large scale environmental sequencing cost effective and keeps the bias small [4]. In Liang et al., 79% of the genetic variations detected by NGS 454 pyrosequencing were not detected by Sanger cloning-sequencing, especially the low-frequency variations (<20% of amplified population) which can be of major significance[5]. However, phylogenetic assignments based on 454 pyrosequencing could be less precise due to shorter read lengths (200 bp) compared to relatively larger sequence lengths resulting from classical 16S rRNA cloning-sequencing (400 bp and above). In the context of anaerobic digestion, 454 pyrosequencing has been utilized in characterizing biogas-producing communities, revealing a number of new bacteria involved [6], [7], but as far as we are aware studying the effect of agitation on entire microbial communities using the technique has rarely been reported.

There were limitations in the previous study [8] with respect to determining the complex communities at different taxonomical hierarchies, as well as identification of dynamics of key microbial populations. One of the main objectives of the present study was to address the gap by characterizing the live microbial consortia through pyrotag sequencing, and to relate the community structures to digestion performance and parameters under agitated and non-agitated conditions, such as CH_4_ production, soluble Chemical Oxygen Demand (sCOD) and Volatile Organic Acids (VOAs) profiles.

## Method and Materials

### Anaerobic digesters operation

Two digesters of 5 L, named digester 1 and 2, were built by modifying Pyrex glass jars [8]. Sugar beet tailings provided by American Crystal Company, Minnesota were used as feedstock for anaerobic digestion. The stored tailings (4°C) were washed with tap water to remove surface residual sugars before loading to the digesters. Both digesters were fed in a batch mode with 0.3 kg (wet weight) of sugar beet tailings and operated at 55°C throughout the experiment.

Digester 1 was operated under non-agitated condition. Two kilograms of bulking materials (lava rocks from landscaping supplier, 0.025 m in average size) were added into digester 1 along with the feedstock to prevent substrate compaction and floatation. Digester 2 was operated under agitated condition without adding the bulking agent. The digester content was continuously mixed at 180 rpm by using a 50.8 mm × 9.5 mm PTFE coated polygon bar and a large volume magnetic stirrer (Bel-Art ScienceWare Cool Stirrer). Two experimental trials were carried out. In trial 1, both digesters were inoculated with 3 L inoculum taken from an anaerobic digester that has been digesting with sugar beet tailings for months. Trial 1 ended at day 18 when the gas production from both digesters was less than 0.05 L@STP L^−1^day^−1^. Digester 1 and 2 were then emptied and washed and residual substrates were discarded. In trial 2, both digesters were fed with fresh 0.3 kg (wet weight) of washed sugar beet tailings and re-inoculated with approximate 3 L of its own sludge liquor recovered from trial 1 (Sludge liquors from digester 1 and 2 were not mixed). Trial 2 ended at day 14 (or day 32 cumulatively) when the gas production from both digesters was less than 0.05 L@STP L^−1^day^−1^.

### Physicochemical Analysis

Total Solids (TS) and Volatile Solids (VS) contents were determined for the feedstock sugar beet tailings [8].

Daily biogas production from the digesters was measured by a positive displacement gas meter. Gas composition (CH_4_ and CO_2_) was analyzed using Fisher Model 1200 Gas Partitioner. sCOD concentration was determined using Hach method 8000. VOA analysis was conducted using Shimadzu gas chromatograph (GC-9AM equipped with a flame ionization detector) for acetic, propionic, isobutyric, butyric, isovaleric and valeric acid concentrations [8].

### Sampling, DNA extraction and PCR

Microbial community analysis was conducted for the original inoculums (day 0), digester 1 liquor and digester 2 liquor. Digester liquors were sampled at day 3, 5 and 18 for trial 1 and day 1, 4, 8, 11, 12 and 14 for trial 2 (or day 19, 22, 26, 29, 30, 32 cumulatively). A total 17 samples were analyzed.

Total DNA was extracted and purified by using FastDNA Kit (MP Biomedicals, Inc., Santa Ana, CA, USA) and PowerClean DNA Clean-up Kit (MO BIO Laboratories, Inc., Carlsbad, CA, USA) respectively, according to the manufacturer's instruction. The quality of DNA was verified by agarose gel electrophoresis. Extracted DNA was stored at −20°C until further use.

For each sample, the V4 hypervariable region of16S rRNA gene was PCR-amplified using the F515/806R primer set that was designed for accurate phylogenetic placement of a broad range of archaeal and bacterial taxa with few biases [9]. The composite forward prime (5′ -GCC TTG CCA GCC CGC TCA GGT GTG CCA GCM GCC GCG GTA A-3′) included the Roche 454-A FLX pyrosequencing adapter (Roche Applied Science, Branford, CT, USA), a two-base linker sequence “GT”, and the primer F515. The composite reverse primer (5′-GCC TCC CTC GCG CCA TCA GNN NNN NNN NNN NGG GGA CTA CVS GGG TAT CTA AT-3′) incorporated the Roche 454-B FLX pyrosequencing adapter, a 12-bp error correcting Golay barcode (designated by NNNNNNNNNNNN), a two-base linker sequence “GG” and the primer 806R. The primer set consisting of 1 forward primer and 12 reverse primers (designated by A1 to A12) that contained a unique barcode to tag each PCR product was used to amply a total of 17 samples in two batches as specified in Table 1. Three independent PCR reactions were carried out for each sample to mitigate reaction level PCR biases. The PCR reaction was performed in a 50 µl volume, containing 20 to 30 ng of DNA template and 20 µL of HotMasterMix [0.5U Taq DNA Polymerase, 45 mM KCl, 2.5 mM Mg^2+^, and 200 µM of dNTP (5 PRIME GmbH, Hilden, Germany)] and 1 µL of barcoded primers (100 pmoles each). The amplification protocol was as follows: initial denaturation at 94°C for 3 minutes, followed by 30 denaturation cycles at 94°C for 45 seconds, annealing at 50°C for 30 seconds, and extension at 65°C for 90 seconds, with a final extension for 10 minutes at 65°C. The tree replicated PCR products were combined for each sample, purified using the QIAquick PCR Purification Kit (Qiagen, Venlo, Limburg, Netherland) and quantified using on-chip gel electrophoresis with Agilent 2100 Bioanalyzer and DNA Lab Chip Kit 7500.

**Table 1:** PCR amplifications of 16s rRNA genes using 12 barcoded primers (an uniform forward primer and 12 different reverse primers A1 through A12).

Cumulative time		Day 0^1^	Day 3	Day 5	Day 18		
Trial 1		Day 0 (inoculum)	Day 3	Day 5	Day 18		
	Digester 1	Primer A1	Primer A2	Primer A4	Primer A6		
	Digester 2	N/A	Primer A3	Primer A5	Primer A7		
**Cumulative time**		**Day 19**	**Day 22^2^**	**Day 26**	**Day 29**	**Day 30**	**Day 32**
**Trial 2**		**Day 1**	**Day 4**	**Day 8**	**Day 11**	**Day 12**	**Day 14**
	Digester 1	Primer A8	Primer A10	Primer A12	N/A	Primer A3	N/A
	Digester 2	Primer A9	Prime A11	Primer A1	Primer A2	Primer A4	Primer A5

1 Sample Day 0 through Day 19 (cumulative time) was amplified in batch 1.

2 Sample Day 22 through Day 32 (cumulative time) was amplified in batch 2.

Because each sample was amplified with a known tagged primer, an equimassic mixture ampicon from different samples could be sequenced simultaneously. Among the total 17 samples, 9 samples were sequenced simultaneously in the first batch, and 8 samples in the second batch. The quantity of each PCR product was made equal within a batch: 432 ng of each PCR product in batch 1 and 480 ng in batch 2.

### Pyrosequencing and analysis

Batch 1 and 2 were sent to Interdisciplinary Center for Biotechnology Research (ICBR) at University of Florida for pyrosequencing using a 454 GS-FLX sequencer (Roche Diagnostics, Co.IN). The raw sequence data were sorted based on the sample specific barcode for batch 1 and 2, respectively, and primer and barcode sequences were then trimmed from the sorted sequences. The trimmed sequences from batch 1 and 2 were then combined and processed through mothur (www.mothur.org/wiki). First, sequences were de-noised and filtered, and chimeric sequences were removed (chimera.unchime) to improve data quality. Second, qualified sequences were clustered to operational taxonomic units (OTUs) defined by a 97% similarity level. Third, diversity analyses and diversity index calculations were performed for Chao1 richness estimation, Good's coverage and rarefaction curves. The variability of community composition between samples was evaluated with Principal Component Analysis (PCA), which is a multivariate ordination method that visually represents distance between samples. More similar communities would be placed closer in the ordination. A similarity matrix of Yue and Clayton (ThetaYC) distances that take into account of both membership and relative abundance was calculated to determine each sample's position in the PCA ordination. Statistical analyses were conducted using the mothur package [10]. Fourth, OTUs were assigned to a taxonomic hierarchy with a confidence threshold of 80% (phylum level at least) according to the mothur modified Ribosomal Data Project (RDP) Release. OTUs with the same taxon were grouped into a phlylotype, which were designated with the taxon name regardless of the taxonomic level.

The sequence data are available from the NCBI Sequence Read Archive (Run SRR1283194).

## Results

### Characteristics of feed substrates

TS and VS contents of sugar beet tailings, loading quantities and packing density were determined for 2 trials and presented in table S1. The average TS and VS contents of sugar beet tailings were 10.9%±0.20% (wt/wt) and 9.7%±0.52% (wt/wt), respectively.

### Methane production

During trial 1, CH_4_ production rate for digester 1 peaked at 0.70 m^3^ d^−1^ (kg VS)^−1^ on day 5, and 0.34 m^3^ d^−1^ (kg VS)^−1^ on day 11 for digester 2. Trial 2 was started by flooding digester 1 and 2 with digester liquor left in trial 1. Digester 1 reached higher CH_4_ production rate during trial 2 than during trial 1 (0.94 m^3^d^−1^(kg VS)^−1^ on day 4), whereas digester 2 exhibited similar production rate (0.35 m^3^d^−1^(kg VS)^−1^ on day 7). Both digesters reached their maximal production rate earlier than during trial 1.

**Figure 1:**
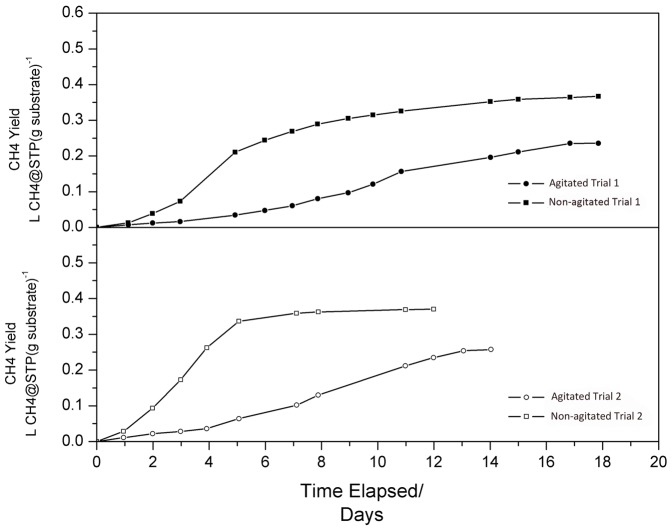
Methane yield of digester 1 and 2.

Cumulative CH_4_ yield for digester 1and 2 in both trials were shown in Figure 1. Digester 1 achieved cumulative CH_4_ yield of 0.37 m^3^ CH_4_ at Standard Temperature and Pressure (STP) (kg VS)^−1^ and digester 2 achieved CH_4_ yield of 0.24 m^3^ CH_4_ at STP (kg VS)^−1^ at the end of trial 1. At the end of trial 2, the CH_4_ yield was 0.35 m^3^ CH4 at STP kg VS^−1^ for digester 1 and 0.21 m^3^ CH4 at STP kg VS^−1^ for digester 2.

### Degradation of organic matters

**Figure 2:**
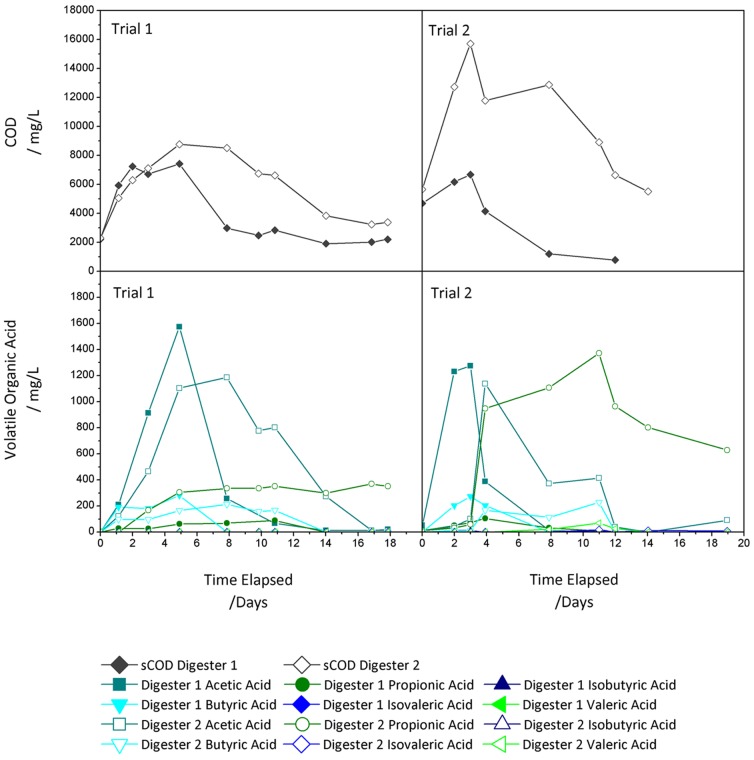
SCOD and VOA profiles of digester 1 and digester 2.

Profiles of soluble COD (sCOD) and VOA concentration of digester 1 and 2 were shown in Figure 2. sCOD concentration of both digesters initially increased, reached a maximum and decreased to a minimum. Digester 1 showed less sCOD accumulation and faster degradation rate than digester 2 in both trials. sCOD degradation began earlier in trial 2 than in trial 1.

The main VOAs detected in both digesters are acetic, butyric and propionic acids. In digester 1, acetic acid was detected with the highest concentrations among VOAs at the beginning of each trial, reaching a maximum around day 3 to 5. Its concentration decreased rapidly to a negligible amount as methane was produced. Similarly, but to a less extent, butyric acid slightly accumulated at the beginning of both trials and then rapidly disappeared from day 4 to 7. Propionic acid was present at constantly low concentration.

In digester 2, the concentration of acetic and butyric acid peaked between day 8 to day 11, and the degradation was delayed compared to that in digester 1. Profiles of propionic acid showed no significant degradation, resulting in an evident accumulation that reached 1000 mg/L in trial 2.

### Microbial community analysis

A total number of 6,993 sequences with average length of 204 bps was obtained from 14,773 raw sequence reads after the quality improving process. The number of sequences for different samples ranged from 126 to 994. Of the total sequences obtained, 149 (2.1% of total sequences) represented lineage from archaea domain. Across 17 samples, 1,137 OTUs (defined at 97% sequence similarity level) were identified. All samples had Good's coverage above 70% and the rarefaction curves were provided in Figure S1. With this level of coverage, the extent of microbial diversity may not have been fully surveyed, but previous work has shown that patterns of beta diversity and overall taxon relative abundances of dominant lineages can be accurately inferred with this depth of sequencing (Bates, Berg-Lyons et al. 2011). The mean Chao1 index at 95% confidence interval (data not shown) indicated a higher microbial richness in digester 1 compared with digester 2 in general. There was a trend that the richness in both digesters increased at first and gradually decreased toward the end of a trial. The decrease in digester 1 was slight, if any, but was significant in digester 2 particular for trial 2.

**Figure 3:**
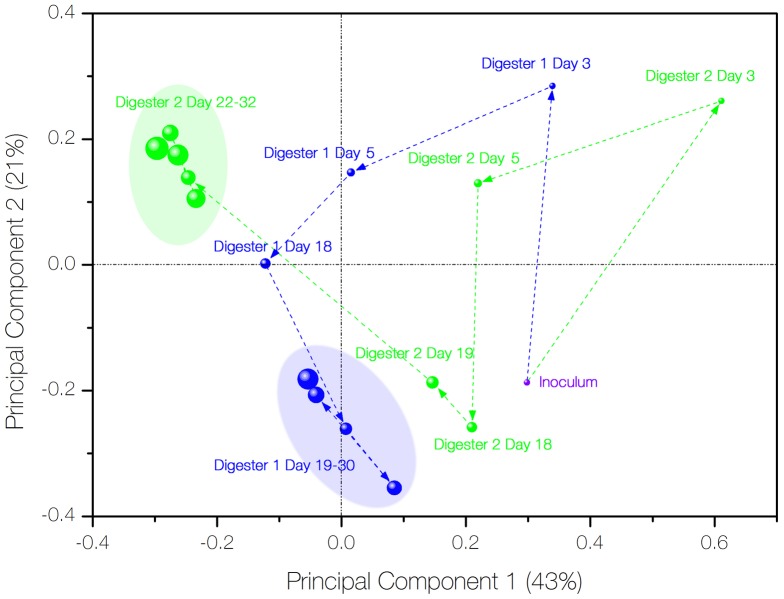
PCA ordination representing variation in the microbial communities based on OTUs retrieved from 17 samples collected from digester 1 and 2. Digester 1 samples are represented by blue spheres; digester 2 samples are represented by green spheres. Samples from the same digester are joined by arrows for indication of time progression; size of spheres indicates the number of days since the start of the experiment.

The variability of community composition was evaluated by a PCA plot (Figure 3). In the analysis, the distance between samples indicated how similar the samples are in terms of community composition. Principal Component 1 and 2 (PC1 and PC2) represented 42% and 23% of the variability in community structure among the samples, respectively. The plot distinguished two clusters: samples of digester 2 from day 22 to day 32 and samples of digester 1 from day 18 to day 30. Samples at early stages of a trial differed significantly in community structure from the original inoculum as well as from each other.

**Figure 4:**
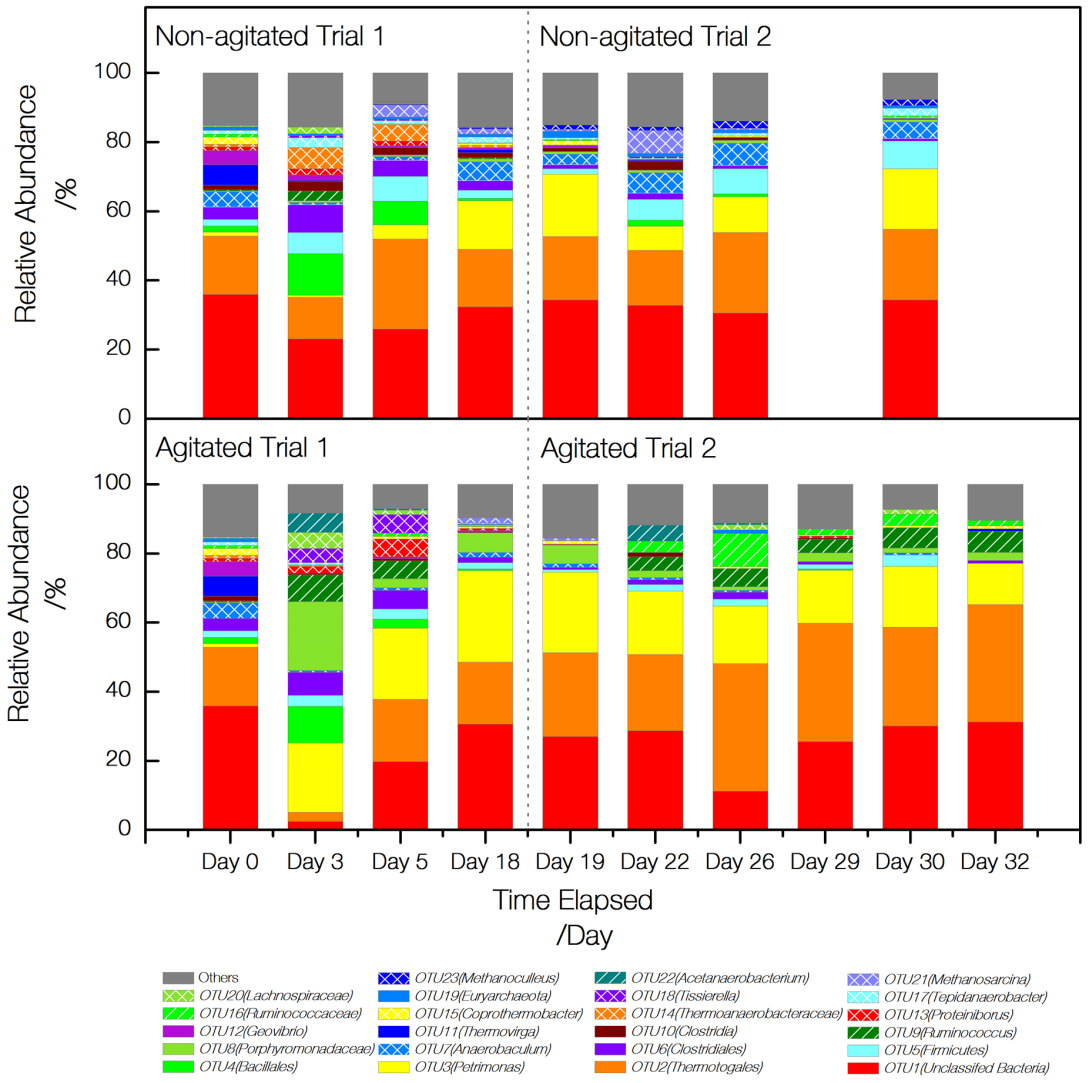
Abundance dynamics of detected phylotypes in digester 1 and digester 2. 23 phlylotypes with highest relative abundance are shown and differentiated by the colors. The remaining phylotypes were pooled into a group called Others. Phylotypes were designated with OTU numbers (OTU1 to OTU23) and the taxon.

1,137 OTUs were taxonomically classified to 117 different phylotypes at similarity threshold of 80%. Top 23 phylotypes with highest relative abundance were selected and analyzed for each sample (Figure 4). The dynamics of relative abundance exhibited the change of community composition over time in a more visual way as compared to Figure 3. An unclassified bacterial and phylotype *Thermotogales* and *Petromonas* were found dominant in the inoculums, digester 1 and digester 2 in general. At beginning of trial 1, the community composition in digester 1 and digester 2 diversified and shifted greatly from that of the inoculums. Phylotype *Bacillaes* gained dominance at day 3 but decreased quickly with progression of the digestion. In trial 2, changes in the community composition were less dynamic.

Despite the aforementioned similarities, digester 1 and 2 differed from each other in many ways with regard to the community compositions. Phylotype *Acetanaerobacterium*, *Ruminococcus* and *Ruminococcaceae* were detected in digester 2 and their relative abundance followed an increasing pattern with development of the digestion. These phylotypes were either not detected or detected at a very low abundance in digester 1. In addition, phylotype *Anaerobaculum* exhibited a different distribution between digester 1 and 2. While *Anaerobaculum* gradually developed and reached relative abundance of 15% in digester 1 (after 15 days), it was identified with constantly low abundance in digester 2.

Archaea were identified at low relative abundance in all samples compared to bacteria. Phylotype *Methanoculleus* and *Methanosarcina* were found abundant among methanogens. In digester 1, the relative abundance of methanogens increased over time, peaking at 5% for trial 1 (day 5) and 8% for trial 2 (day 22), respectively. In contrast, the development of methanogens in digester 2 was slow and the relative abundance never exceeded 1.4% (day 18) in trial 1. It continuously reduced in trial 2 until no significant detection was obtained near the trial end. Selected phyloptyes (including *Desulfotomaculum*, *Pelotomaculum* and *Syntrophomonas* that was not shown in Figure 4) were grouped at phylum, order and genus levels to reveal a clearer picture of the community composition at different taxonomic hierarchies (Table S2).

## Discussion

### Digestion performance comparison

The non-agitated digester 1 achieved higher CH_4_ yield and CH_4_ production rate than the agitated digester 2. In trial 2, digester 1 showed higher sCOD degradation rate and CH_4_ production rate than in trial 2 due to that the digester was inoculated with liquor recovered from the previous trial, which had been adapted to tailings decomposition. Digester 2 was also inoculated with adapted liquor from trial 1, but neither CH_4_ production nor substrate decomposition showed accelerated rate. This suggested that the agitation affected digester 2 performance adversely.

The VOA profiles revealed an initial accumulation of acetic acid in both digesters, but it rapidly disappeared in digester 1 while it persisted for 10 to 12 days before degradation in digester 2. Conversion of propionic acid appeared problematic in digester 2 as high accumulation was observed, especially for trial 2. In anaerobic digestion, acetogenic bacteria ferment propionate to acetate which is then utilized by acetotrophic methanogens to produce CH_4_. The high accumulation of propionic acid in digester 2 probably suggested the inhibition of propionate degradation. The accumulation was further increased in trial 2, indicating digestion inhibition could be exacerbated if the digester liquor was exposed to agitating and used for inoculation continuously. It is well accepted that accumulation of propionic acid indicates an anaerobic process instability [11], but it can also be considered as the cause of the process failure as its accumulation has been reported inhibitory for methanogens activity [12].

### Difference in microbial community composition

Digester 1 and digester 2 clearly varied in their bacterial and archaeal community compositions as the PCA ordination indicated. The operating conditions of the two digesters only differed in agitation status, suggesting agitation could have a strong influence driving microbial community of anaerobic digestion. Though both digesters were dominated by phylotype *Thermotogales*, *Petrimonas* and an unclassified bacterium, the separation between the digesters was likely linked to the different distribution of some less dominant but important organisms. Methanogens and *Anaerobaculum* related species were mostly characterized in digester 1, whereas *Acetanaerobacterium*, *Ruminococcus* and *Ruminococcaceae* related species were prevalent in digester 2.

The PCA plot formed two main clusters: digester 1 samples from day 19 to day 30, and digester 2 samples from day 22 to day 32, which were retrieved from trial 2. On the contrary, samples from trial 1 were highly variable. This is an indication of the progress that microbial populations shifted substantially from the inoculum community in response to the onset of operation and progressively adapted to tailings decomposition as the operation continued in trial 2. The tighter clustering of digester 2 (day 22 to day 32) suggested a lower microbial diversity as verified by the Chao1 richness estimation (results not shown) in comparison with digester 1. Interestingly, the community evolving between digester 1 and 2 seemed to follow a similar path from day 0 to day 5 but subsequently developed in separate ways to disparate compositions. This could imply the effect of agitation was not instantaneous but rather cumulative.

#### Microbial community composition and digestion performance

16s rDNA sequences reads were compared to the entries of RDP database, and assigned to phylogeneic groups. However, a large portion of sequences were classified to an unclassified phylotype, suggesting the complexity of microbial communities in anaerobic digestion is yet to be characterized. Among identified phylotypes, *Anaerobaculum* has been known to degrade peptide and a limited number of carbohydrates [13]–[15]. Sugihara et al studied the propionate-degrading ability of a microbial consortium exposed to periodic propionate pulses in sequencing fed batch reactor and reported an *Anaerobaculum*-related species being dominant [16]. Phylotype *Anaerobaculum* seemed to play a role in propionate degradation, even though it has not been recognized as a syntrophic propionate utilizing bacterium. It can be postulated that the low abundance of *Anaerobaculum* in digester 2 probably resulted in accumulation of propionic acid.

Archaeal (mostly methanogens) versus bacterial community ratio generally agreed with the reported methanogen proportions ranging from 0.1% to 15% of the total microbial population [17], [18]. Identified methanogens were closely related to *Methanoculleus* and *Methanosarcina* species. Members of *Methanoculleus* are hydrogenotrophic methanogens [19], while *Methanosarcina* species are mostly acetoclasic but also able to use H_2_ [20]. In digester 1, sequences originating from phylotype *Methanosarcina* were generally more abundant than from *Methanoculleus.* The relative abundance of *Methanosarcina* was seen to follow a dynamic that coincided with the digestion performance. The marked increase from day 3 to day 5 in trial 1 and day 1 to day 4 in trial 2 corresponded to the high CH_4_ production rate and the significant reduction of acetic acid during a similar time frame. *Methanosarcina* spp. has been reported to have higher growth rates and tolerance to pH changes and could potentially lead to stable methenogensis in anaerobic digestion [21]. It should be noted there was an almost complete lack of methanogens in digester 2 throughout trial 2. This does not necessarily imply they are absent, but indicated a fairly low abundance of these organism compared with those in digester 1. It was, however, correlated to the low CH_4_ production and the accumulation of propionic acid in digester 2 particular for trial 2.

Phylotypes *Acetanaerobacterium*, *Ruminococcus* and *Ruminococcaceae* were found with high relative abundance in digester 2. Those species were closely related and all belonged to family *Ruminococcaceae*, which are known to degrade cellulose and produce hydrogen (H_2_) as one of the fermentation products [22]–[24]. Application of *Ruminococcus* species has been widely used for H_2_ production from a variety of feedstock[25], [26]. Interestingly, some species of *Ruminococcus* were reported to produce propionate other than ethanol as fermentation products, which could also lead to propionic acid accumulation [27].

#### Possible functions of selected organisms

In anaerobic digestion, hydrogen could be generated through fermentation of intermediate products as sugars or VOAs [28]. However, hydrogen (H_2_) production is energetically unfavorable due to proton being a poor electron acceptor. The development of syntrophic communities allows H_2_ production to become energetically favorable and sustain degradation of organic compounds and production of CH_4_. Due to that syntrophic metabolism, methanogenic activity has to be suppressed in order to produce free H_2_ or it would have been readily converted to CH_4_ in anaerobic digestion. pH control is desirable for H_2_ production because methanogenic activity drops sharply in an acidic environment [29].However, production of free H_2_ at low partial pressure has been reported in anaerobic processes operated at neural or near neutral pH [30], [31], which is similar to the operational condition of digester 2 (pH 7.2±0.2). It was speculated that *Ruminococcus* related bacteria produced free H_2_ in digester 2 even though it was typically considered energetically unfavorable. Some studies seemed to support this speculation. Rychlik and May investigated the effect of *Methanobrevibacter smithii* on growth rate, organic acid production and specific ATP activity of *Ruminococcus albus* in the co-ulture [32]. The result indicated no increase in the growth rate, acetate or ATP production, suggesting *Ruminococcus albus* did not receive energetic advantage from co-culturing with the methanogen and the syntrophic metabolism was not preferred. Zhou et al investigated the effect of methanogenic inhibitors on methaneogens and three rumen bacteria *Fibrobacter succinogenes, Ruminococcus albus* and *Ruminococcus flavefaciens* [33]. While the anti-methanogen compounds effectively reduced the population of methanogens, the inhibiting effect was insignificant or none on the bacterial population, suggesting the syntrophic relation was weak or did not exist. It appeared that, unlike typical hydrogen producing bacteria, phylotype *Ruminococcus* may not always rely on the syntrophic relationship with methanogens to grow and produced free H_2_ in digester 2. Its concentration was expected to be very low that was not analyzed in biogas composition, but may have exerted enough inhibition on methanogensis as discussed below.

Phylotypes *Desulfotomaculum*, *Pelotomaculum* and *Syntrophomonas* were detected in both digester 1 and 2 at low proportion (see Table S2). Species in these genera are well known as obligate syntrophic bacteria that play crucial role in the degradation of short chain fatty acid such as propionate and butyrate [34], [35]. The lower CH_4_ yield and the low abundance of methanogens in digester 2 may be attributed to the extreme sensitivity of obligate syntrophic bacteria to H_2_. Even at low partial pressure, H_2_ can inhibit syntrophic metabolism and thereby limit the substrate supply to methanogens. This inhibition seemed to be cumulative as the methanogen abundance decreased with time. Inhibition of syntrophic interactions also result in accumulations of VOAs, as suggested by the high accumulation of propionic acid observed for digester 2, particularly in trial 2. Menes and Muxi [14] reported H_2_ inhibition on glucose utilization by *Anaerobaculum mobile*, which could explain the low abundance of phylotype *Anaerobaculum* phylotype in digester 2.

## Conclusion

The study was conducted to investigate the effect of agitating on anaerobic digestion, focusing on the microbial community dynamics that was revealed to be heavily influenced by agitation. While methanogens and syntrophic bacteria were identified at low relative abundance, species related to *Acetanaerobacterium*, *Ruminococcus* and *Ruminococcaceae* that were known to produce H2 through sugar fermentation were abundant in the agitated digester. The affected digestion performance under agitation may be inferred that the presence of minor amount of H_2_ inhibited the syntrophic interaction and VOAs degradation.

## Supporting Information

Figure S1 OTUs based rarefaction curves of inoculum, digester 1 and digester 2.
(TIF)

Table S1 Substrate characteristic and loading quantities for digester 1 and 2.
(DOCX)

Table S2 Taxonomic composition of digester 1 and digester 2 at the rank of phylum, order and genus.
(DOCX)
