# Supplementary figures and images for: Microbial Ecology in Anaerobic Digestion at Agitated and Non-Agitated Conditions
Source: PLoS One. 2014 Oct 14;9(10):e109769. doi: 10.1371/journal.pone.0109769 (PMC4196933; doi:10.1371/journal.pone.0109769)

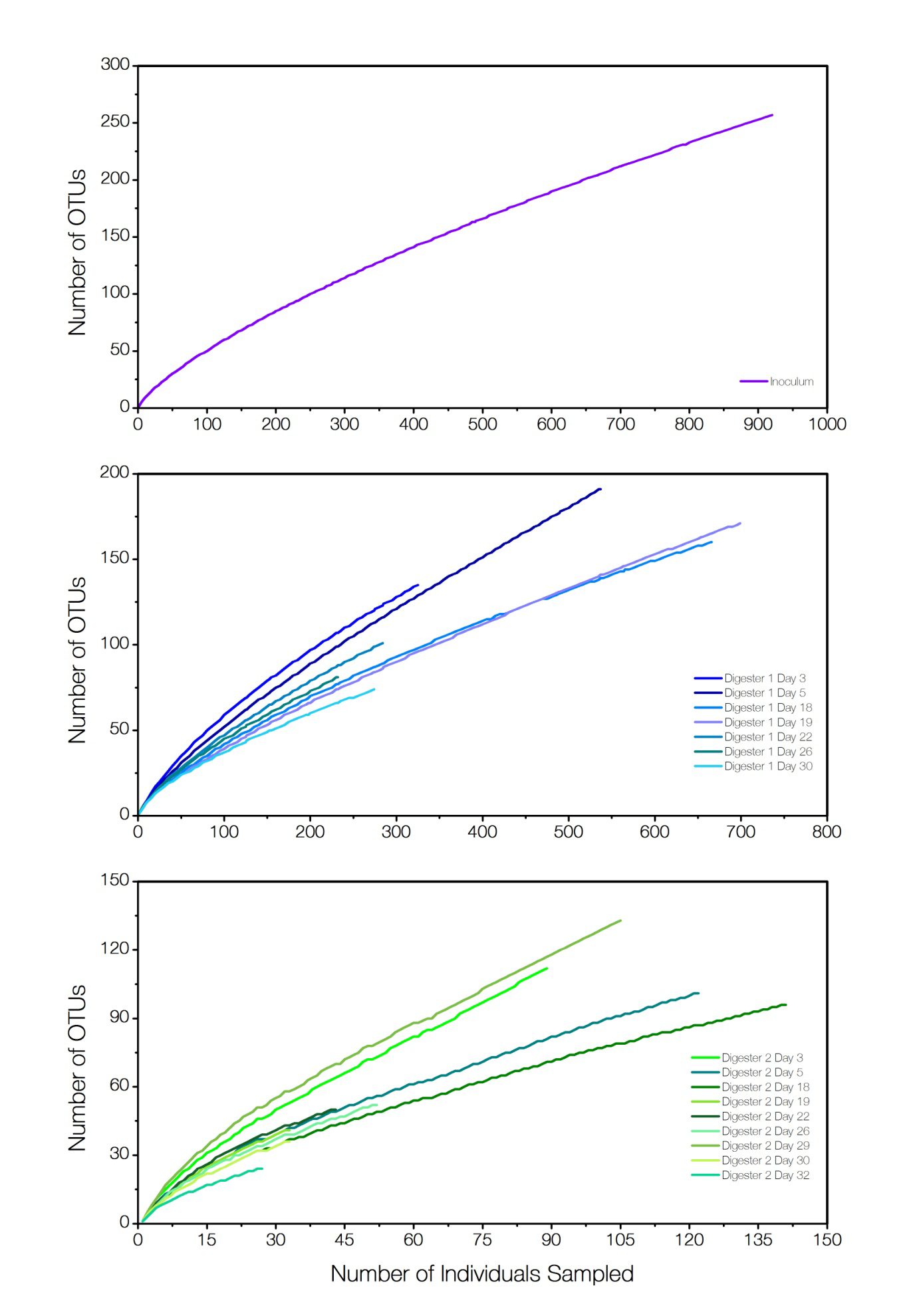

Supplement: Figure S1 — OTUs based rarefaction curves of inoculum, digester 1 and digester 2. (TIF) [file pone.0109769.s001.tif]
