# Supplementary material for: Microbial Ecology in Anaerobic Digestion at Agitated and Non-Agitated Conditions
Source: PLoS One. 2014 Oct 14;9(10):e109769. doi: 10.1371/journal.pone.0109769 (PMC4196933; doi:10.1371/journal.pone.0109769)
Supplement: Table S1 — Substrate characteristic and loading quantities for digester 1 and 2. (DOCX) [file pone.0109769.s002.docx]

Table S1. Substrate characteristic and loading quantities for digester 1 and 2

|  | Unit | Trial 1 | Trial 2 |
| --- | --- | --- | --- |
| TS | % (wt/wt) | 10.91%±0.20% | 10.90%±0.35% |
| VS | % (wt/wt) | 9.39%±0.75% | 9.94%±0.31% |
| Wet weight | kg | 0.3 | 0.3 |
| Dry weight | kg | 0.033 | 0.033 |
| Volatile matter | kg | 0.028 | 0.030 |
| Inoculumn added | L | 3 | 3 |
| Packing density | kg/m^3^,wet wt basis | 100 | 100 |
| Packing density | kg/m^3^, dry wt basis | 10.91 | 10.9 |
