# Supplementary material for: Microbial Ecology in Anaerobic Digestion at Agitated and Non-Agitated Conditions
Source: PLoS One. 2014 Oct 14;9(10):e109769. doi: 10.1371/journal.pone.0109769 (PMC4196933; doi:10.1371/journal.pone.0109769)
Supplement: Table S2 — Taxonomic composition of digester 1 and digester 2 at the rank of phylum, order and genus. (DOCX) [file pone.0109769.s003.docx]

Table S2. Taxonomic composition of digester 1 and digester 2 at the rank of phylum, order and genus

| Time | Day 0 | Day 3 | | Day 5 | | Day 18 | | Day 19 | | Day 22 | | Day 26 | | Day 29 | Day 30 | | Day 32 |
| --- | --- | --- | --- | --- | --- | --- | --- | --- | --- | --- | --- | --- | --- | --- | --- | --- | --- |
|  | ^1^I | ^2^D1 | ^3^D2 | D1 | D2 | D1 | D2 | D1 | D2 | D1 | D2 | D1 | D2 | D2 | D1 | D2 | D2 |
| *^4^Bacteria* | ^7^71.03 | 66.48 | 74.47 | 72.81 | 78.22 | 79.64 | 87.51 | 80.46 | 86.61 | 68.92 | 87.46 | 85.01 | 85.84 | 86.68 | 83.86 | 86.67 | 90.54 |
| *Unclassifed* | 26.23 | 13.55 | 2.06 | 16.47 | 20.28 | 32.36 | 31.34 | 34.11 | 27.36 | 26.17 | 29.38 | 31.22 | 11.31 | 26.21 | 24.31 | 30.06 | 31.27 |
| *^5^Bacteroidetes* | 1.31 | 0.68 | 30.06 | 4.46 | 23.16 | 14.86 | 32.09 | 18.74 | 28.68 | 7.74 | 20.27 | 11.21 | 17.68 | 16.87 | 18.25 | 18.95 | 14.28 |
| *^6^Bacteroidales* | 1.31 | 0.68 | 30.06 | 4.46 | 23.16 | 14.86 | 32.09 | 18.74 | 28.68 | 7.74 | 20.27 | 11.21 | 17.68 | 16.87 | 18.25 | 18.95 | 14.28 |
| *^8^Petrimonas* | 1.11 | 0.06 | 10.04 | 4.09 | 20.48 | 13.96 | 26.42 | 18.02 | 23.33 | 7.04 | 18.31 | 10.35 | 16.67 | 14.32 | 17.52 | 17.64 | 11.90 |
| *Deferribacteres* | 4.12 | 1.54 | 0.00 | 0.56 | 0.49 | 0.60 | 0.35 | 0.72 | 0.00 | 0.35 | 0.00 | 0.00 | 0.00 | 0.00 | 0.00 | 0.00 | 0.00 |
| *Deferribacterales* | 4.12 | 1.54 | 0.00 | 0.56 | 0.49 | 0.60 | 0.35 | 0.72 | 0.00 | 0.35 | 0.00 | 0.00 | 0.00 | 0.00 | 0.00 | 0.00 | 0.00 |
| *Geovibrio* | 4.12 | 1.54 | 0.00 | 0.56 | 0.49 | 0.60 | 0.35 | 0.72 | 0.00 | 0.35 | 0.00 | 0.00 | 0.00 | 0.00 | 0.00 | 0.00 | 0.00 |
| *Firmcutes* | 12.10 | 37.78 | 49.28 | 24.13 | 25.51 | 8.84 | 5.91 | 5.43 | 5.34 | 12.48 | 15.14 | 12.85 | 19.48 | 9.35 | 13.76 | 8.50 | 9.28 |
| *Bacillales* | 1.82 | 12.00 | 10.65 | 6.89 | 2.68 | 0.76 | 0.53 | 0.00 | 0.00 | 1.76 | 0.00 | 0.86 | 0.00 | 0.42 | 0.00 | 0.00 | 0.00 |
| *Clostridiales* | 4.04 | 10.71 | 24.95 | 3.28 | 18.44 | 2.98 | 2.90 | 1.42 | 3.50 | 2.27 | 11.87 | 3.37 | 16.95 | 6.80 | 5.36 | 4.58 | 8.49 |
| *Ruminococcus* | 0.30 | 2.77 | 7.77 | 0.19 | 5.12 | 0.30 | 0.00 | 0.00 | 3.10 | 0.00 | 4.02 | 0.00 | 5.33 | 3.87 | 0.00 | 5..92 | 6.74 |
| *Acetanaerobacterium* | 0.00 | 0.00 | 5.52 | 0.00 | 0.49 | 0.00 | 0.18 | 0.00 | 0.00 | 0.00 | 4.58 | 0.00 | 0.51 | 0.00 | 0.00 | 0.00 | 0.00 |
| *Proteiniborus* | 1.01 | 1.85 | 2.05 | 1.30 | 5.12 | 0.00 | 0.71 | 0.00 | 0.15 | 0.00 | 0.00 | 0.00 | 0.00 | 0.43 | 0.00 | 0.00 | 0.00 |
| *Tepidanaerobacter* | 1.01 | 2.77 | 0.61 | 0.93 | 0.00 | 1.65 | 0.18 | 0.57 | 0.00 | 0.35 | 0.00 | 0.86 | 0.00 | 0.00 | 2.19 | 0.00 | 0.00 |
| *Tissierella* | 0.20 | 0.62 | 4.09 | 0.37 | 5.37 | 0.00 | 0.18 | 0.00 | 0.00 | 0.35 | 0.00 | 0.00 | 0.00 | 0.00 | 0.00 | 0.00 | 0.00 |
| *Desulfotomaculum* | 0.07 | 0.85 | 0.00 | 0.15 | 0.00 | 0.00 | 0.00 | 0.00 | 0.00 | 0.94 | 0.00 | 0.00 | 0.00 | 0.00 | 0.40 | 0.00 | 0.16 |
| *Pelotomaculum* | 0.00 | 0.00 | 0.00 | 0.15 | 0.00 | 0.00 | 0.00 | 0.19 | 0.15 | 0.00 | 0.00 | 0.00 | 0.00 | 0.40 | 0.00 | 0.00 | 0.00 |
| *Syntrophomonas* | 0.34 | 0.00 | 0.00 | 0.00 | 0.14 | 0.88 | 1.12 | 0.37 | 0.10 | 0.63 | 0.00 | 2.01 | 0.00 | 0.40 | 2.40 | 0.00 | 0.00 |
| *Thermoanaerobacterales* | 2.92 | 6.15 | 0.20 | 4.84 | 0.73 | 1.20 | 0.53 | 1.43 | 0.92 | 0.00 | 0.00 | 0.43 | 0.51 | 0.43 | 0.37 | 0.65 | 0.79 |
| *Coprothermobacter* | 2.11 | 0.00 | 0.20 | 0.19 | 0.73 | 0.60 | 0.53 | 1.29 | 0.92 | 0.00 | 0.00 | 0.43 | 0.51 | 0.43 | 0.37 | 0.65 | 0.79 |
| *Synergistetes* | 10.36 | 0.93 | 0.41 | 1.11 | 0.73 | 6.31 | 1.4 | 3.14 | 1.07 | 6.34 | 0.65 | 6.45 | 0.51 | 0 | 7.11 | 0.6 | 0.79 |
| *Synergistales* | 10.36 | 0.93 | 0.41 | 1.11 | 0.73 | 6.31 | 1.4 | 3.14 | 1.07 | 6.34 | 0.65 | 6.45 | 0.51 | 0 | 7.11 | 0.6 | 0.79 |
| *Anaerobaculum* | 4.52 | 0.62 | 0.41 | 1.11 | 0.73 | 5.41 | 1.40 | 3.14 | 1.07 | 5.99 | 0.65 | 6.45 | 0.51 | 0.00 | 6.74 | 0.60 | 0.00 |
| *Thermovirga* | 5.84 | 0.31 | 0.00 | 0.00 | 0.00 | 0.90 | 0.00 | 0.00 | 0.00 | 0.35 | 0.00 | 0.00 | 0.00 | 0.00 | 0.37 | 0.00 | 0.79 |
| *Thermotogae* | 16.91 | 12.00 | 2.66 | 26.08 | 18.05 | 16.67 | 26.42 | 18.32 | 24.16 | 15.84 | 22.02 | 23.28 | 36.86 | 34.25 | 20.43 | 28.56 | 34.92 |
| *Thermotogales* | 16.91 | 12.00 | 2.66 | 26.08 | 18.05 | 16.67 | 26.42 | 18.32 | 24.16 | 15.84 | 32.02 | 23.28 | 36.86 | 34.25 | 20.43 | 28.56 | 34.92 |
| *^4^Archeae* | 0.91 | 0.62 | 0.00 | 4.85 | 0.00 | 2.80 | 1.60 | 3.65 | 0.91 | 8.69 | 0.00 | 3.45 | 1.01 | 0.00 | 2.55 | 0.00 | 0.00 |
| *^5^Euryarchaeota* | 0.91 | 0.62 | 0.00 | 4.85 | 0.00 | 2.80 | 1.60 | 3.65 | 0.91 | 8.69 | 0.00 | 3.45 | 1.01 | 0.00 | 2.55 | 0.00 | 0.00 |
| *^6^Methanomicrobiales* | 0.00 | 0.00 | 0.00 | 1.19 | 0.00 | 0.45 | 0.00 | 1.29 | 0.15 | 1.11 | 0.00 | 2.16 | 0.00 | 0.00 | 1.82 | 0.00 | 0.00 |
| *^8^Methanoculleus* | 0.00 | 0.00 | 0.00 | 1.19 | 0.00 | 0.45 | 0.00 | 1.29 | 0.15 | 1.11 | 0.00 | 2.16 | 0.00 | 0.00 | 1.82 | 0.00 | 0.00 |
| *Methanosarcinales* | 0.00 | 0.00 | 0.00 | 3.42 | 0.00 | 1.45 | 1.42 | 0.50 | 0.61 | 6.52 | 0.00 | 0.43 | 0.00 | 0.00 | 0.00 | 0.00 | 0.00 |
| *Methanosarcina* | 0.00 | 0.00 | 0.00 | 3.42 | 0.00 | 1.45 | 1.42 | 0.05 | 0.61 | 6.52 | 0.00 | 0.43 | 0.00 | 0.00 | 0.00 | 0.00 | 0.00 |

^1^Inoculum .^2^Digester 1, ^3^Digester 2, ^4^Taxonomic domains are shaded in dark grey.

^5^Bacterial and archaeal phyla are shaded in grey ^.^

^6^Bacterial and archaeal orders are not shaded.

^7^Relative abundance are calculated as a phylogenenic group in percentage of the total phylotypes identified

^8^Bacterial and archaeal genera and their relative abundance are shown in grey
